# Ecological connectivity assessment in a strongly structured fire salamander (*Salamandra salamandra*) population

**DOI:** 10.1002/ece3.1617

**Published:** 2015-07-27

**Authors:** Luciano Bani, Giulia Pisa, Massimiliano Luppi, Giulia Spilotros, Elena Fabbri, Ettore Randi, Valerio Orioli

**Affiliations:** 1 Department of Earth and Environmental Sciences, University of Milano-BicoccaPiazza della Scienza 1, I-20126, Milano, Italy; 2 Department of Biology, University of Milanovia Celoria 26, I-20133, Milano, Italy; 3 Laboratory of Genetics, Istituto Superiore per la Protezione e la Ricerca Ambientale (ISPRA)I-40064, Ozzano Emilia, Bologna, Italy; 4 Section of Biology and Environmental Science, Department of Biotechnology, Chemistry and Environmental Engineering, Aalborg UniversitySohngaardsholmsvej 57, DK-9000, Aalborg, Denmark

**Keywords:** CIRCUITSCAPE, fragmented populations, isolation by distance, isolation by resistance, landscape genetic, MaxEnt, molecular markers

## Abstract

Small populations are more prone to extinction if the dispersal among them is not adequately maintained by ecological connections. The degree of isolation between populations could be evaluated measuring their genetic distance, which depends on the respective geographic (isolation by distance, IBD) and/or ecological (isolation by resistance, IBR) distances. The aim of this study was to assess the ecological connectivity of fire salamander *Salamandra salamandra* populations by means of a landscape genetic approach. The species lives in broad-leaved forest ecosystems and is particularly affected by fragmentation due to its habitat selectivity and low dispersal capability. We analyzed 477 biological samples collected in 47 sampling locations (SLs) in the mainly continuous populations of the Prealpine and Eastern foothill lowland (PEF) and 10 SLs in the fragmented populations of the Western foothill (WF) lowland of Lombardy (northern Italy). Pairwise genetic distances (Chord distance, D_C_) were estimated from allele frequencies of 16 microsatellites loci. Ecological distances were calculated using one of the most promising methodology in landscape genetics studies, the circuit theory, applied to habitat suitability maps. We realized two habitat suitability models: one without barriers (EcoD) and a second one accounting for the possible barrier effect of main roads (EcoD_b_). Mantel tests between distance matrices highlighted how the Log-D_C_ in PEF populations was related to log-transformed geographic distance (confirming a prevalence of IBD), while it was explained by the Log-EcoD, and particularly by the Log-EcoD_b_, in WF populations, even when accounting for the confounding effect of geographic distance (highlighting a prevalence of IBR). Moreover, we also demonstrated how considering the overall population, the effect of Euclidean or ecological distances on genetic distances acting at the level of a single group (PEF or WF populations) could not be detected, when population are strongly structured.

## Introduction

The importance of biodiversity is recognized since long time (Costanza et al. 1997; Chapin et al. 2000; Hooper et al. 2005), but, until now, the rate of biodiversity loss has not been reduced because anthropic pressures on natural ecosystems still persist and are even intensified. Although habitat destruction is one of the most detrimental effects, its effects still remain largely unknown (but see Wilcove et al. 1998; Primack 2010; Groom et al. 2006; Kareiva and Marvier 2011). Actually, its overall effects remain complex to understand, as it triggers many other processes, which play cumulatively or interactively with it, determining the dynamics and the fate of populations (Gilpin and Soulé 1986; Lindenmayer 1995; Young et al. 1996). For example, the habitat loss (physical loss) and degradation (loss of ecological functionality) produce a reduction of population size. Small populations become more prone to extinction because of genetic drift, inbreeding, environmental and demographic stochasticity, falling in the extinction vortex (Gilpin and Soulé 1986).

Habitat loss usually determines a fragmentation process of the original population, affecting the spatial distribution of the remaining subpopulations, confining them to residual habitat fragments. Subpopulations may constitute a typical meta-population, when dispersal between them persists (sensu Levins 1969), or several isolated small populations, when dispersal is halted. Indeed, the isolation prevents the genetic exchange between subpopulations, determining their genetic differentiation and emphasizing the negative effects previously produced by the habitat loss.

For these reasons, population survival in fragmented landscapes depends on the conservation or restoration of functional (i.e., ecological) connectivity among fragments of residual habitats, which should be guaranteed by the presence of ecological corridors. They allow maintaining the ecological connection between subpopulations, which remains bounded by dispersal (Hanski and Simberloff 1997), in a meta-population form (Levins 1969). The knowledge of the ecology of fragmented populations is thus essential in order to prevent and mitigate the effects of habitat isolation (Saunders et al. 1987; Burgman and Lindenmayer 1998). Understanding how landscape features affect dispersal between populations is thus important for both conservation purposes and evolutionary processes (Moore et al. 2011).

Traditional approaches aiming to evaluate the effectiveness of dispersal between populations, like radiotracking or capture–mark–recapture methods (White and Garrott 1990; Barrett and Peles 1999; Fagan and Calabrese 2006) appear to be not completely adequate to evaluate the ecological connectivity, because they hardly detect movements from birth sites to reproductive ones. However, these movements can be assessed using DNA molecular markers that are able to detect gene flow in a meta-population (Cushman 2006). For this reason, molecular markers are now one of the most efficient and promising tools to verify the ecological connectivity (Avise 1994; Frankham et al. 2002; Frankham 2006). Among molecular markers, hypervariable microsatellites (or short tandem repeats, STRs) are widely used in landscape genetic studies for several practical reasons, as they are now available for many taxa, and they require the collection of small amounts of tissue and a relatively limited field effort.

Modelling genetic pattern over large areas using environmental spatial data as covariates (i.e., landscape genetic; Manel et al. 2003; Holderegger and Wagner 2006; Manel and Segelbacher 2009), may lead to quantitate how landscape features shape population genetic variability more accurately than traditional ecological methods do. The landscape genetic approach is ideal for investigating ecological connectivity, particularly in heterogeneous landscapes (Holderegger and Wagner 2008; Moore et al. 2011).

The pairwise correlation analysis between genetic distance and Euclidean distance (ED) (assuming a spatially homogenous landscape) or between genetic distance and ecological distance (taking into account the influence of a heterogeneous landscape) in populations are two of the most commonly adopted approaches in landscape genetic for evaluating the importance of organism–environment interaction regarding the gene flow (Dixon et al. 2006; Epps et al. 2007; Spear et al. 2010; Storfer et al. 2010). The IBD theory (IBD; Wright 1943) predicts that genetic similarity among individuals decreases as the ED between them increases: this pattern results from spatially limited dispersal, as individuals living nearby to one another are more likely to interbreed than geographically distant ones. However, straight-line geographic distances assumed by IBD theory may not adequately describe the true pattern of dispersal, especially in fragmented landscapes where the presence of barriers or a low permeable environmental matrix could strongly affect the gene flow between populations. In these cases, several recent studies have demonstrated that measures of distance, reflecting landscape resistance (i.e., Ecological distance), often explain a greater proportion of the genetic variability than simple ED (Michels et al. 2001; Coulon et al. 2004; Spear et al. 2005; Vignieri 2005; Broquet et al. 2006; Cushman et al. 2006; Stevens et al. 2006; Pérez-Espona et al. 2008; Schwartz et al. 2009; Goldberg and Waits 2010). This concept is the core of the IBR theory (IBR; McRae 2006).

Pairwise ecological distances between populations are often estimated by means of the ecological resistance approach (e.g., Bani et al. 2002; Adriaensen et al. 2003; Beier et al. 2006, 2009; Tracey 2006; Compton et al. 2007; Carroll et al. 2011) that assess the different effects played by land-use and landscape features on possible dispersal movements and thus on genetic distances (Cushman et al. 2006; Shirk et al. 2010; Sork and Waits 2010; Spear et al. 2010). These effects are usually quantified by habitat suitability models, which allow calculating the landscape permeability or resistance, affecting dispersal between populations. The potential pathways along which dispersal may occur can be identified by several methods based on permeability or resistance maps. The most traditional method is the least-cost path analysis (LCPA; e.g., Bani et al. 2002; Adriaensen et al. 2003), which calculates a unique pathway between two locations resulting in the least accumulated resistance. Recently, McRae et al. (2008) developed the circuit theory analysis, which compares the pathways along which dispersal may occur with an electrical circuit. According to this theory, the landscape matrix and barriers affect individuals flow as the circuit resistance affects the amount of current flowing through an electrical circuit. This approach improves the LCPA, by identifying multiple pathways connecting populations.

The main aim of this study was to evaluate the ecological connectivity of strongly structured fire salamander *Salamandra salamandra* (*AMPHIBIA*, *URODELA*) populations using molecular markers. The species is widely distributed in the broad-leaved forest ecosystems of the study area (Lanza et al. 2007). Moreover, although the species may have a high migratory activity (Schmidt et al. 2007; but also Schmidt et al. 2014), it is characterized by a low dispersal capability (Denoël 1996; Schulte et al. 2007) and thus particularly threatened by habitat fragmentation. We chose microsatellites as molecular markers, because they are widely used for evaluating the effective genetic connectivity between populations (Avise 1994; Frankham et al. 2002; Frankham 2006) and they have already been identified for the fire salamander in discrete numbers (Steinfartz et al. 2004; Hendrix et al. 2010). The study area is located in the Prealpine and foothill belt of Lombardy (northern Italy), where, according to a hierarchical genetic structure analysis, fire salamander populations were divided in two main groups (Pisa et al. 2015). The first one inhabits a portion of the Western foothill (WF) belt, where residual forest patches are almost completely isolated by the urban sprawl. The second group corresponds to the populations sampled in the mostly continuous forest areas of the Prealpine and Eastern foothill lowland (PEF). These last sampling populations were almost genetically homogeneous, while those of the WF group appeared strongly structured. In order to estimate landscape resistance to gene flow, we developed two habitat suitability models, considering land-use classes and morphology only, or accounting also for the possible effects played by barriers on dispersal. Then, according to the circuit theory approach, we used the two habitat suitability models to measure the ecological distances among populations. We tested the effectiveness of IBD (using ED) or IBR (using ecological distance) theories in explaining the variability of the genetic distance measured among sampling populations. This analysis was performed separately for the two groups, PEF and WF, in order to assess which theory, IBD or IBR, better explain the genetic distances among sampling locations (SLs). On the base of Pisa et al. (2015), we expected that in the PEF group the genetic distances should be related to ED (following the IBD theory), while in the WF group, the effect of ecological distances should prevails (following the IBR theory). Moreover, we abovementioned analysis was performed on all SLs on the whole, in order to verify if the effect of Euclidean or ecological distances on genetic distances, acting at the level of a single group (PEF and WF populations), could hidden, when population are strongly structured.

## Methods

### Study area, sampling design, and tissue sampling

**Figure 1:**
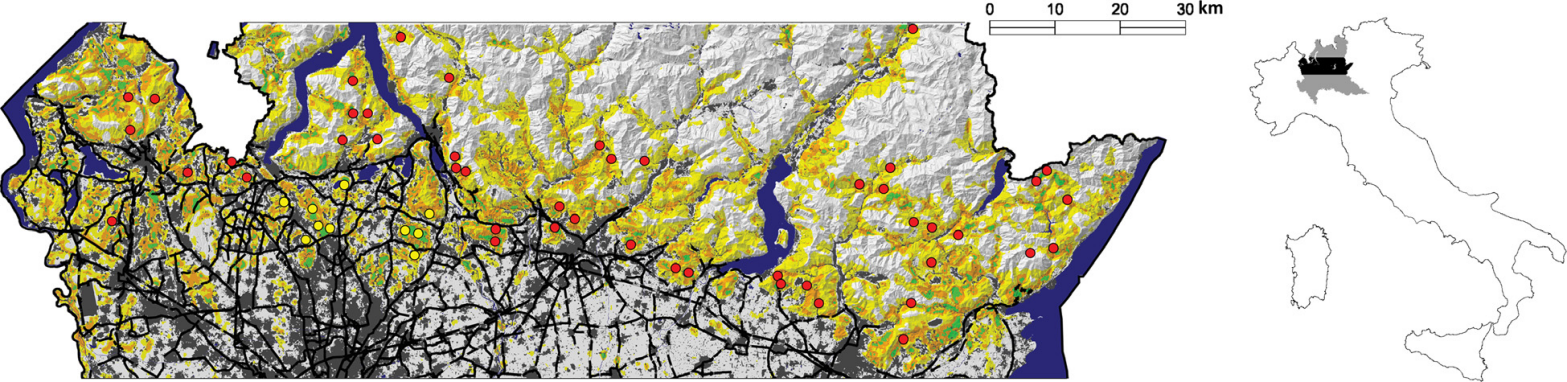
Study area in Lombardy (northern Italy). Map of the whole life-cycle habitat suitability model realized with MaxEnt (hill shade from Digital Elevation Model); Green areas: fire salamander presence probability (FSpp) >50%; Orange areas: FSpp 25–50%; Yellow areas: FSpp 5–25%. Blue: main water bodies. Black lines: main roads. Red dots: sampling locations (SLs) assigned to the Prealpine and Eastern foothill group; Yellow dots: SLs assigned to the Western foothill group (Pisa et al. 2015).

The study area is a belt of about 8000 km^2^, corresponding to the main range of fire salamander within the Prealpine and foothill areas of Lombardy (northern Italy, Fig. 1). Sampling design, described in Pisa et al. (2015), was realized in order to collect a representative sample of the species population in the study area. According to it, we explored potential breeding sites (Di Cerbo and Razzetti 2004), looking for salamander larvae along streams and their meanders located in forest areas. We collected biological samples, year-round from 2010 to 2013, in 174 breeding sites grouped in 57 SLs (Table S1). In each breeding site, we sampled from 1 to 4 fire salamander larvae cutting 3–4 mm of tissue from their tail, for a total of 477 tissue samples. The collected samples were stored in ethanol and at −20°C until DNA extraction. For further details on sampling design and tissue collection, see Pisa et al. (2015).

### Genetic distance

Genetic data came from a previous study on fire salamander population structure performed by Pisa et al. (2015). The authors genotyped 477 individuals, using 16 microsatellite loci (Steinfartz et al. 2004; Hendrix et al. 2010), identifying a hierarchical genetic population structure of the sampled populations: two main clusters were identified, one corresponding to the Prealpine area (PEF group), which maintain genetic connection with some Eastern foothill populations, and another cluster located in the WF area (WF group). Even if the first group, with 47 SLs, is distributed over a wide area, it appeared poorly structured. Conversely, the second group, with only 10 SLs, appeared to be strongly structured in four clusters, although it was geographically restricted in a relatively narrow area.

In order to analyze the genetic spatial variation of individuals, we assessed the spatial autocorrelation of allele frequencies of genotyped individuals, using the Moran's I correlogram. The method assumes each individual as a population, and thus, allele frequency for a specific allele is 1 if the individual is homozygous, 0.5 if heterozygous, 0 otherwise (Shimatani and Takahashi 2003). Moran's I calculated at the individual level represents an estimate, at different distance class, of the Wright's coefficient of relationship ρ (Hardy and Vekemans 1999). The shortest distance at which the Moran's I is not significantly different from zero represents the extent within which individuals are genetically similar. We compared the genetic spatial variation of individuals pertaining to the PEF and WF groups computing the correlogram for each of them separately using GenAlEx v. 6.501 (Peakall and Smouse 2006, 2012).

To estimate the genetic distance between each pair of our populations we chose the chord distance (D_C_; Cavalli-Sforza and Edwards 1967). This measure seems to be more robust than the commonly used *F*_ST_, as it may reflect allele frequencies changes more rapidly (Kalinowski 2002), and thus, it is more appropriate to account for genetic drift, particularly at fine geographic scale (Goldberg and Waits 2010). We calculated the pairwise D_C_, using the software Microsatellite Analyzer (Dieringer and Schlötterer 2002), for all the 57 SLs pooled together (ALL group), and for the two main subpopulations separately, that is the 47 SLs of the PEF group and the 10 SLs of the WF group.

### Ecological distances

#### Habitat suitability models

In the study area, Pisa et al. (2015) collected only presence data of fire salamander larvae. Salamander data were georeferenced and thus associated to a digital cartography from which environmental variables were extracted. To build the habitat suitability model for the species, we chose MaxEnt (Maximum Entropy modelling; Phillips et al. 2005), one of the most used software analyzing presence-only data. This software evaluates how a predefined set of environmental variables may affect the probability of the presence of a studied species, comparing variable values in the presence sites with those of a large set of randomly extracted pseudo-absence points (e.g., 10,000).

The effectiveness of a habitat suitability model depends on the scale at which environmental variables affect the modeled phase of the species life cycle (Trani 2002; Mateo Sánchez et al. 2013). In fragmented landscapes, animals tend to disperse in suitable habitats, avoiding the most hostiles (Baguette and Van Dyck 2007). Thus, when a habitat suitability model is specifically realized to evaluate ecological connectivity, it should not only account for suitable breeding or home-range habitats, but also for dispersal ones. This purpose can be achieved measuring the covariates at a spatial scale corresponding to the magnitude of species dispersal.

As the fire salamander requires different habitats during its life cycle, we first developed a breeding habitat model (aquatic phase), able to identify suitable reproductive areas. Secondly, we built a whole life-cycle model (encompassing from the aquatic to dispersal phase). The two steps approach was required because the whole life-cycle suitable areas were identified only around the previously identified suitable breeding ones. Moreover, the modelling could not be done in a single step as the modeled areas of the two phases differ in scale: the first model (breeding model) was developed only for the breeding study area, containing all potential suitable areas for the deposition of larvae, while the second one (whole life-cycle model) for all areas around the breeding ones. The whole life-cycle area extent was as wide as the dispersal species capability from breeding areas.

The breeding study area was defined as a 30-m buffer from both sides of all cartographic streams in forest areas, according to the sampling design criteria. As cartographic streams are digitalized as linear elements, and not as areal, the buffer width was designed in order to account for the actual stream surface, considering the maximum distance (about 30 m) between cartographic linear streams and the sampled breeding sites.

The breeding habitat model was developed considering as sampling units, our 174 georeferenced breeding sites (presence points) and 10,000 pseudo-absence points, randomly extracted in the breeding study area. We assumed that environmental variables might affect the suitability of breeding sites in a 30 m radius around them. For this reason, we considered as covariates the stream order (Strahler method; Strahler 1957) of the closest digitalized stream and, in a 30 m radius around each point, the fractional cover of the four main land use classes (all but forests, from the digital land use map of Lombardy, with 10 m pixel size derived from DUSAF 2.1 -1:10,000; ERSAF 2010): urban areas, farmlands, grasslands, and shrublands. We also considered the mean of elevation, slope, and aspect, calculated in the 30 m radius (Table 1). The output of the breeding suitability model was the presence probability of the salamander larvae in the breeding study area.

**Table 1:** Ranges of covariates used in habitat suitability models and variable contribution estimated by MaxEnt for the (a) breeding and (b) whole life-cycle model (see Supplementary material for MaxEnt outputs)

	Variable	Variable range (presence sites)	Variable range (random points)	Contribution (%)	Permutation importance
(a)	Elevation	246–1502	68–2010	32.4	41.9
	Slope	0–42.12	0–77.31	29.2	23.8
	Urban areas	0–0.4	0–1	10.1	11.8
	Farmlands	0–0.8	0–1	10.4	7.4
	Stream order	1–4	1–8	5.6	4.7
	Grasslands	0–1	0–1	4.3	4.7
	Shrublands	0–0	0–1	4.7	2.6
	Aspect (W-E)	−0.80–0.67	−1–1	1.5	1.8
	Aspect (S-N)	−0.84–0.91	−1–1	1.9	1.3
(b)	Broad-leaved forests	0–1	0–1	13.9	22
	Mixed forests	0–0.94	0–1	5.0	13.5
	Breeding site suitability	0.01–0.34	0–0.39	39.9	10.3
	Elevation	242–1562	75–2049	4.5	9.8
	Coniferous forests	0–0.2	0–1	7.6	7.9
	Road density	0–0.28	0–0.57	6.4	6.5
	Shrubs	0–0.14	0–0.87	4.1	6.3
	Grasslands	0–0.56	0–0.93	1.8	6.0
	Urban areas	0–0.35	0–0.98	5.2	5.7
	Slope	1.71–38.72	0–48.49	3.9	5.0
	Farmlands	0–0.58	0–0.97	6.7	4.9
	Aspect (S-N)	−0.42–0.05	−0.84–0.4	0.7	2.0
	Aspect (W-E)	−0.04–0.26	−0.31–0.54	0.3	0.2

The whole life-cycle model was built for a larger area around suitable breeding areas using the same sampling units of the previous model. To realize an appropriate habitat suitability model encompassing the whole species life-cycle, including the dispersal phase (i.e., postnatal dispersal), we measured covariates in a radius of 500 m around breeding sites. This measure should include the postnatal dispersal distance of the fire salamander in suitable habitats (Joly 1968; Denoël 1996; Schulte et al. 2007). Thus, the whole life-cycle study area extended 500 m from the sampling units. As covariates, we considered the mean breeding habitat suitability (i.e., the presence probability of the salamander larvae estimated by the first model), the land-use fractional cover (urban areas, farmlands, grasslands, broad-leaved forests, mixed forests, coniferous forests, and shrublands), all roads density, and the mean of elevation, slope, and aspect (Table 1). The output of this model was a habitat suitability map corresponding to the surface probability of the salamander presence for its whole life cycle.

In addition, we realized a second whole life-cycle habitat suitability map, derived from the previous one, penalizing by a factor of 1/100 the salamander presence probability in all those areas covered by main ground-level roads or continuous urban areas. As we considered these anthropic features as potential barriers to salamander movements, this model should better account for their effects on dispersal, which in turn affects the ecological connectivity among populations. We considered this second approach useful because, although the first model account for the whole life-cycle suitability for the species in its range within the study areas, it could underestimate the possible effect of sharp barriers among potential suitable areas that cannot be detected by the habitat suitability only.

### CIRCUITSCAPE analysis

In order to evaluate the degree of connections between populations, we used the CIRCUITSCAPE software (Shah and McRae 2008) that allows to evaluate the ecological distance between each pair of populations in a heterogeneous landscape using the circuit theory. This ecological distance incorporates both the concepts of minimum movement distance and the availability of alternative pathways connecting each pair of populations, and its value decreases as more connections are added. Circuit resistance (*R*, ohm) can be interpreted as an ecological resistance, corresponding to the opposition played by each landscape element or feature to the movement of an organism during dispersal. As ecological resistance surface, the CIRCUITSCAPE software uses the inverse of a habitat suitability map. The ecological distance is the effective resistance, Ȓ, calculated as the ratio of the voltage between two populations and currents flowing along the several pathways connecting them (McRae et al. 2008). Thus, the estimated ecological distance between each pair of populations is a measure of their isolation.

Ecological distances were calculated, using the pairwise mode, for the ALL, PEF, and WF group and for the two habitat suitability models, with (EcoD_b_) and without (EcoD) barriers.

### IBD and IBR tests

In order to test IBD and IBR theories, we built three kind of triangular distance matrices for the chord (D_C_; Table S2), Euclidean (ED; Table S3), and ecological (EcoD [Table S4] or EcoD_b_ [Table S5]) distances, containing the respective distances calculated for each pair of SLs. To analyze the correlation among distance matrices, we used simple Mantel test (Mantel 1967; Mantel and Valand 1970) and partial Mantel test (Smouse et al. 1986), which are considered effective in spatial analysis of genetic data (Legendre and Fortin 2010). We first tested the IBD theory calculating the simple Mantel correlation coefficient (*r*_M_) between D_C_ and ED. We tested the IBR theory calculating the *r*_M_ between D_C_ and EcoD or EcoD_b_. Finally, we calculated the partial Mantel correlation coefficient between D_C_ and the two ecological distances controlling for the ED effect. We performed all the analyses for the ALL, PEF, and WF group. As the Mantel test results could be biased by the nonlinear relationships between distances, we performed analyses using both the original untransformed and log-transformed genetic, Euclidean, and ecological distance matrices (Slatkin 1993; Rousset 1997; McRae 2006; Liu et al. 2012; Phillipsen and Lytle 2012; Ruiz-González et al. 2014). All tests were based on 10,000 permutations. Simple and partial Mantel tests were performed by the software R 3.0.0 (R Core Team 2013) with the *ecodist* package (Goslee and Urban 2007). Using the *phytools* package (Revell 2012) in R, we drew scatter plots aiming to illustrate evidences of IBD and IBR theories emerging from Mantel tests, plotting D_C_ against ED (for simple Mantel tests) and D_C_ against the residuals of simple Mantel tests between ecological distances and ED (for partial Mantel tests).

## Results

### Genetic and Euclidean distances

**Figure 2:**
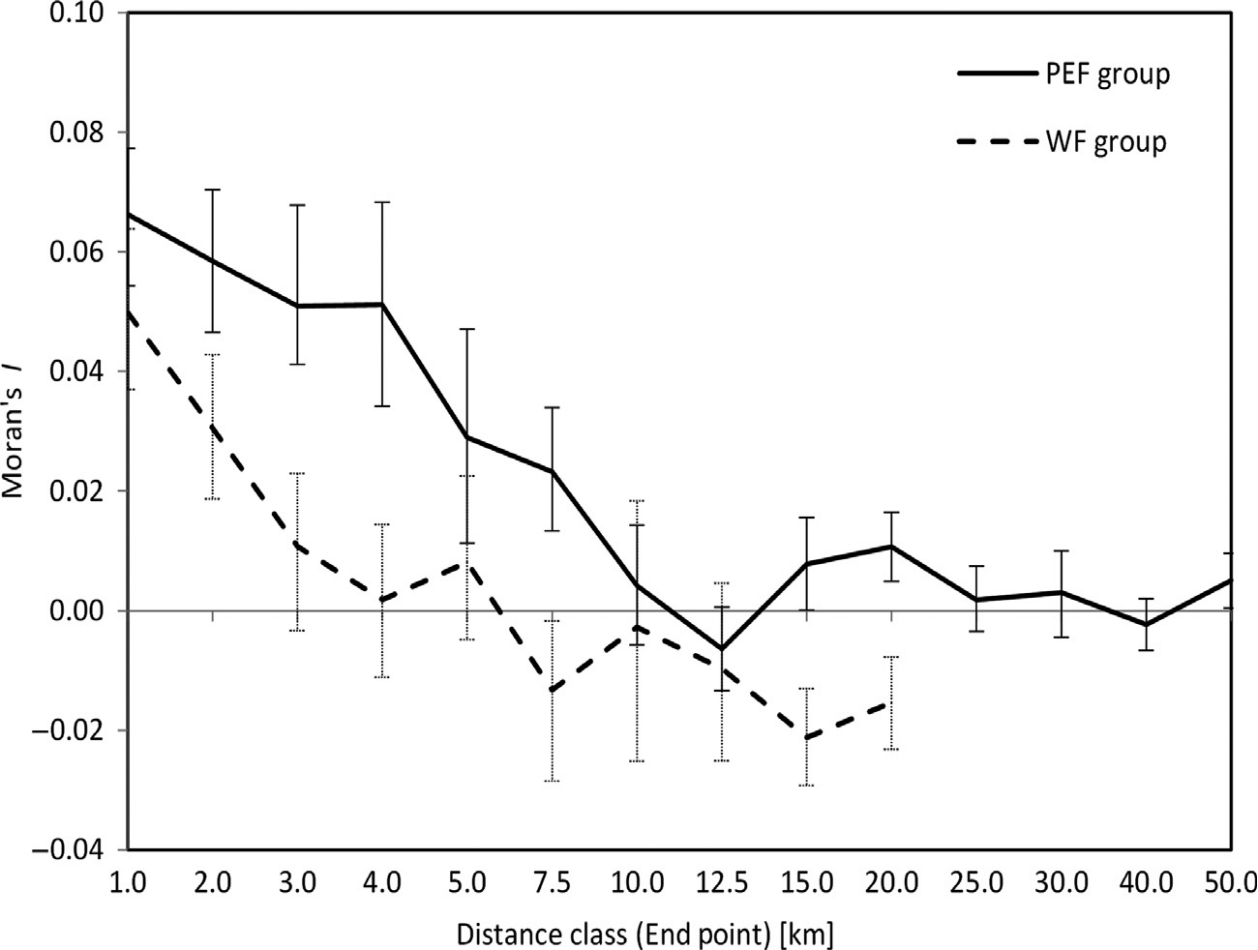
Moran's I spatial autocorrelogram estimated using multilocus genotype and Euclidean distances for the Prealpine and Eastern foothill (solid line) and Western foothill (dashed line) group. Error bars indicate 95% confidence intervals.

Moran’ I was calculated for the PEF and WF group, in 14 or 10 variable distance classes, respectively. The number of distance classes was chosen in order to cover all the extent of the WF group and at least 50 km for the PEF group. This choice allowed us to more easily compare the differences between the two groups. Both groups showed a decline of Moran's I with distance, but with a different slope. The Moran's I estimated for the PEF group resulted not significantly different from zero at 10 km, while the WF group at only 3 km (Fig. 2). Moreover, the estimated values of the WF group were significantly lower from those of the PEF group as far as 7.5 km.

Chord distance values ranged from 0.193 to 0.567 (mean 0.341, SD 0.061) for the ALL group, from 0.197 to 0.552 (mean 0.341, SD 0.059) for the PEF group, and from 0.193 to 0.480 (mean 0.312, SD 0.076) for the WF group (Table S2). The ED varied from 1.3 to 146.0 km for the ALL and PEF group (ALL: mean 52.7, SD 33.9; PEF: mean 54.4, SD 34.6) and from 1.8 to 22.3 km (mean 11.1, SD 6.1) for the WF group (Table S3).

### Ecological distances

The breeding habitat suitability model, developed with MaxEnt, showed a good predictive ability (AUC = 0.828). Moreover, the threshold to balance sensitivity and specificity of the model equaled 0.450, which allowed a correct classification of about 75% of the sampling units, being the training omission rate 0.246. We assessed the covariates contribution according to the permutation importance, which is recognized as a robust method to ascertain the independent variables prominence (Phillips and Dudik 2008). The most important environmental variables in a radius of 30 m from breeding sites were elevation (41.9% of permutation importance), slope (23.8%), and the fractional cover of urban areas (11.8%) (Table 1). The breeding suitability decreased when elevation increased, and the larvae presence probability approaches zero at 1500 m a.s.l. The optimal slope ranged between about 5° and 20°, while the presence probability was close to zero for slope higher than 40°, where runoff should be too strong to permit slow-flowing pool formation. Urban areas showed an almost linear negative effect on the presence probability of salamander larvae.

The whole life-cycle model showed a good predictive ability as well (AUC = 0.879) and the threshold value to balance sensitivity and specificity was 0.368. This implies a correct classification of about 80% of the sampling units, being the training omission rate 0.201. The most important variables affecting salamander presence probability in a radius of 500 m from breeding sites were the fractional cover of broad-leaved (22.0% of permutation importance) and mixed forests (13.5%) and the breeding site suitability (10.3%; Table 1). All these variables positively affected the probability of the presence of salamanders. The whole life-cycle habitat suitability map of the fire salamander is shown in Figure1. The ecological distances were calculated applying CIRCUITSCAPE on the output map of the whole life-cycle habitat suitability model (used as conductance map). In the cumulative map of currents (Fig. S1), the areas with high current intensity represent the possible pathways expected to be more permeable to dispersal. The EcoD ranged from 1.3 to 210.6 (mean 81.8, SD 48.5) for the ALL group, from 1.4 to 210.2 (mean 82.6, SD 52.4) for the PEF group, and from 1.3 to 97.3 (mean 35.4, SD 27.2) for the WF group (Table S4). The EcoD_b_ extended from 1.3 to 1371.9 (mean 233.8, SD 212.1) for the ALL group, from 1.5 to 1371.9 (mean 243.6, SD 236.2) for the PEF group, and from 1.3 to 151.9 (mean 62.1, SD 42.8) for the WF group (Table S5).

### IBD and IBR tests

**Figure 3:**
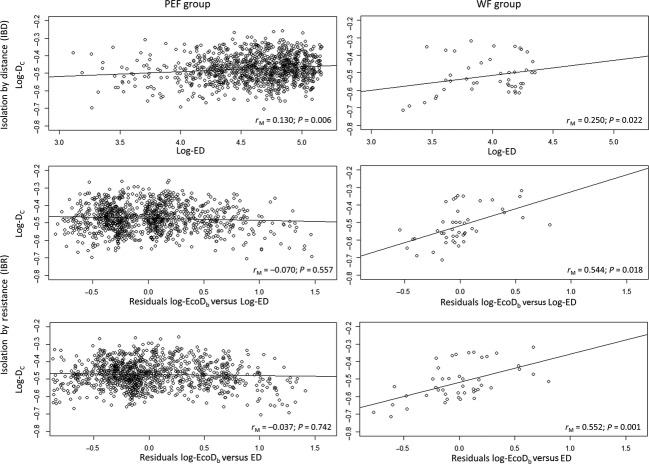
Isolation by distance (IBD) and isolation by resistance (IBR), scatter plots. Above, log-transformed chord distance (Log-D_C_) versus log-transformed Euclidean distance (Log-ED). In the middle, Log-D_C_ versus residuals between ecological distance with barriers (Log-EcoD_b_) and Log-ED. Below, Log-D_C_ versus residuals between Log-EcoD_b_ and Euclidean distance (ED). On the left, scatter plots of the Prealpine and Eastern foothill group; on the right, scatter plots of the Western foothill group. *r*_M_, Mantel test correlation coefficient; *P*, Mantel test significance (see Table 2).

None of the tests of IBD and IBR theories on D_C_, without logarithm transformation on distance matrices, produced significant results (Table 2a). Considering both log-transformed chord and EDs, IBD theory was always proved effective for ALL, PEF, and WF group (*P* < 0.022; Table 2b; Fig. 3), while regarding untransformed ED, IBD theory resulted significant only for all SL pooled together (ALL group, *P* < 0.029; Table 2c). IBR theory was tested on log-transformed matrices, accounting also for the confounding effect of geographic distance. Indeed, as expected, geographic distances (ED and Log-ED) were strongly (*r*_M_ > 0.527) and significantly related (*P* < 0.001) to ecological distances (EcoD, EcoD_b_, Log-EcoD and Log-EcoD_b_). Testing IBR for the ALL and PEF group only simple Mantel test between Log-D_C_ and Log-EcoD resulted significant (*P* < 0.040), but accounting for the effect of geographic distance (ED or Log-ED), the partial Mantel tests were not significant (Table 2b,c; Fig. 3). Conversely, the IBR test on the WF group resulted always significant, except for the partial Mantel test between log-D_C_ and Log-EcoD ¦ Log-ED (*P* = 0.094). Among the significant Mantel tests of the WF group, the highest *r*_M_ values were obtained for the Log-EcoD_b_ ¦ ED (*r*_M_ = 0.552; *P* = 0.001; Table 2c; Fig. 3) and Log-EcoD_b_ ¦ Log-ED (*r*_M_ = 0.544; *P* = 0.018; Table 2b; Fig. 3).

**Table 2:** Mantel correlation coefficients (*r*_M_) and their significance (*P*) between (a) all untransformed and (b) all log-transformed distance matrices (c) log-transformed genetic and ecological distance matrices and untransformed ED matrix

	Isolation	Type of	ALL group		PEF group		WF group	
	Theory tested	Mantel test	r_M_	P	r_M_	P	r_M_	P
(a) D_C_								
ED	IBD	S	0.117	0.053	0.096	0.141	0.146	0.306
EcoD	IBR	S	0.140	0.051	0.098	0.259	0.331	0.105
EcoD ¦ ED	IBR	P	0.078	0.468	0.031	0.787	0.326	0.155
EcoD_b_	IBR	S	−0.061	0.559	−0.104	0.391	0.310	0.105
EcoD_b_ ¦ ED	IBR	P	−0.107	0.341	−0.153	0.230	0.377	0.149
(b) Log-D_C_								
Log-ED	IBD	S	0.153	0.001	0.130	0.006	0.258	0.022
Log-EcoD	IBR	S	0.187	0.001	0.140	0.040	0.474	0.018
Log-EcoD ¦ Log-ED	IBR	P	0.108	0.258	0.059	0.572	0.492	0.094
Log-EcoD_b_	IBR	S	0.082	0.298	0.033	0.729	0.478	0.002
Log-EcoD_b_ ¦ Log-ED	IBR	P	−0.012	0.919	−0.070	0.557	0.544	0.018
(c) Log-D_C_								
ED	IBD	S	0.128	0.029	0.104	0.115	0.193	0.147
Log-EcoD ¦ ED	IBR	P	0.137	0.091	0.095	0.278	0.522	0.039
Log-EcoD_b_ ¦ ED	IBR	P	0.017	0.858	−0.037	0.742	0.552	0.001

IBD, isolation by distance theory; IBR, isolation by resistance theory; S, simple Mantel test; P, partial Mantel test; D_C_, chord distance; ED, Euclidean distance; EcoD, ecological distance without barriers; EcoD_b_, ecological distance with barriers. We performed tests for all sampling locations pooled together (ALL group) and for the Prealpine and Eastern foothill lowland (PEF group) and Western foothill (WF) lowland (WF group) separately. The number of permutation was set to 10,000.

## Discussion

A previous work, that analyzed the genetic population str ucture of the fire salamander in our study area, showed a sharply separation between PEF lowland populations (PEF) from those inhabiting a portion of the WF lowland. Moreover, populations of the WF group, beyond being ecologically separated from those of the PEF group, tended also to be isolated from each other (Pisa et al. 2015). This genetic population structure was confirmed and further investigated by the present research, which allowed identifying and quantitating some significant anthropogenic features, such as land use and roads, in separating salamander populations. Indeed, as shown in the Figure1 by the whole life-cycle habitat suitability map, the 10 SLs of the WF group, were located in a highly urbanized foothill lowland, and most of them appeared to be surrounded by very low suitable areas and separated from each other by relevant barriers. Conversely, most of the foothill PEF populations seemed to be more connected with the Prealpine belt. This different condition between WF and PEF populations was also highlighted by the spatial autocorrelogram, which showed how genetic similarity among individuals resulted sharply different in extent in the two groups. Indeed, the Moran's I showed a prevalence of gene flow over relative large distances (and thus IBD) in the PEF group, while a strong genetic population structure over short distance (and thus a preeminent IBR) in the WF group (Fig. 2).

In continuous habitats, individuals living nearby to one another are more likely to interbreed than geographically distant ones (according to IBD theory), but this could not be true in fragmented landscapes, where the presence of low permeable matrix and/or barriers alter the canonical pattern of dispersal process (according to IBR theory). This phenomenon may heavily affect the genetic structure of populations, even at short distances. In fact, although the range of genetic distances among sampling population was similar in the PEF and WF group, the span of ED of the PEF group was about one order of magnitude higher than that of the WF group.

The whole life-cycle habitat suitability model here realized seemed to highlight the patchy pattern of matrix permeability in the area inhabited by the WF populations. Here, low suitable or unsuitable habitats for the species dominate, in consequence to both an extensive presence of anthropic land uses and a thick network of infrastructural barriers. The usefulness of considering the presence of barriers, beyond the pure habitat suitability, allowed us to incorporate the effect played by main roads, otherwise not adequately accounted for in the whole life-cycle habitat suitability model. This is the reason why we decided to penalize the value of this habitat suitability model in the case of the presence of important infrastructural barriers that possibly impeded salamander movement and thus disrupted the dispersal process.

The conceptual basis of the IBR model lies in the analogous properties of gene flow in deme networks and conductance in electronic circuits (McRae 2006); thus, the whole life-cycle habitat suitability model was used as a conductance surface. The effective ecological resistance calculated for each pairs of SLs (i.e., ecological distance or ecological distance with barriers), using CIRCUITSCAPE, was related to their genetic distance, controlling for the geographic distance (i.e., the possible superimposed effect of IBD). As in real landscapes dispersal usually occur in two dimensions, the genetic differentiation increases with the logarithm of distance. Indeed, the resistance to dispersal, following both the IBD or IBR theory, increases with the logarithm of Euclidean or ecological distances in two-dimensional conductors, as well as are the environmental matrices (McRae 2006). The results of our Mantel tests were completely in agreement with this dispersal model.

On the whole (ALL group), the IBD theory alone was proved effective in explaining the genetic distance among populations. Indeed, as the ED, and particularly its logarithm, increased among SLs, the higher was the logarithm of D_C_ among them (*r*_M_ = 0.153, *P* = 0.001). This was also true for the PEF group (*r*_M_ = 0.130, *P* = 0.006), where the forest cover is mainly continuous and the presence of barriers less severe, allowing to conserve a more permeable environmental matrix. In fact, in this group, the effect of the ecological distance (Log-EcoD) is quite similar to that of the Euclidean distance (Log-ED). In addition, in the PEF group, no barrier effect resulted significant, highlighting the irrelevant role of main roads. Conversely, the genetic distance seemed less affected by the geographic distance in the WF group, but rather by the resistance opposed by the matrix and barriers, according to the IBR theory. Although in the WF group, the relationship between Log-D_C_ and Log-ED was significant (*r*_M_ = 0.258, *P* = 0.022), the Mantel's r between Log-D_C_ and Log-EcoD_b_, controlling for the superimposed effect of the Log-ED or simply ED, amounted to 0.544 (*P* = 0.018) or 0.552 (*P* = 0.001), respectively. These last Mantel's r were quite high values and may be explained by the strong structure already found by Pisa et al. (2015) within the WF group. Indeed, WF populations resulted separated from each other by a densely populated continuous urban area, and several main roads with a high vehicular traffic, which are difficult to be crossed by salamanders. Habitat remnants are thus confined in small areas by anthropic surfaces, which hinder or halt the dispersal processes (Gibbs 1998; Vos and Chardon 1998; deMaynadier and Hunter 2000; Carr and Fahrig 2001) or increase mortality (Fahrig et al. 1995; Carr and Fahrig 2001). All these phenomena act in synergy in emphasizing genetic divergence among populations and even genetic diversity reduction (Reh and Seitz 1990; Pisa et al. 2015). Conversely, the presence of main roads and continuous urban areas seemed not to play a significant role in the PEF group, probably due to a generally lower vehicular traffic in the hilly area and also for the absence of large conurbations. Moreover, even the presence of large and uncrossable natural barriers (as large lakes) that separate continuous extensive suitable areas for the species in the hilly area did not cause a general population differentiation in the PEF group. This is likely due to the persistence of large populations in this areas, avoiding high levels of genetic structuring, as instead occurs in sampling population of the WF group (see Pisa et al. 2015). Finally, if the low permeable matrix and barriers play a fundamental role in separating sampling populations pertaining to the WF group, it is easy to expect that the separation between WF and PEF group could be due to a barrier effect in itself even higher, caused by the intensive urban sprawl and the dense high traffic road network present in this area.

From a methodological point of view, the results of the present research emphasized the effectiveness of the circuit theory approach to define the ecological distances in fragmented populations. By means of this method, we identified significant correlations between genetic and ecological distances. Moreover, we highlight the usefulness of ecological conductance maps (i.e., habitat suitability maps) as a basic tool for objectively determining the causes of some important conservation problems (Dixo et al. 2009; Carroll et al. 2011). In addition, the cumulative current map (Figure S1) indicates the more permeable pathways along which dispersal may occur, allowing identifying the most important potential ecological corridors for the conservation of population connectivity. In conclusion, the adopted method proved effective in identifying conservation issues deriving from habitat isolation, one of the most important factors affecting population persistence and even disrupting adaptive and evolutionary processes (Templeton et al. 2001) in fragmented landscapes. In particular, it emphasizes the importance to analyze separately subpopulations pertaining to a strongly structured population within which the determinants of genetic differentiation may be explained by IBD or IBR theory. In this way is thus possible to ascertain whether the genetic differentiation is locally due to the ED among populations or to the presence of a low permeable landscape or barriers that could lead to the isolation of populations.

## Supporting Information

**Figure S1**. Cumulative map of currents according habitat suitability model with barriers, realized with CIRCUITSCAPE software.

**Table S1**. Sampling populations coordinates.

**Table S2**. Distance matrices between populations: Genetic distance matrix.

**Table S3**. Distance matrices between populations: Euclidean distance matrix.

**Table S4**. Distance matrices between populations: Ecological distance matrix without barriers.

**Table S5**. Distance matrices between populations: Ecological distance matrix with barriers.

**Data S1.** Output of MaxEnt models.
