# Supplementary figures and images for: Ecological connectivity assessment in a strongly structured fire salamander (Salamandra salamandra) population
Source: Ecol Evol. 2015 Jul 27;5(16):3472–85. doi: 10.1002/ece3.1617 (PMC4569041; doi:10.1002/ece3.1617)

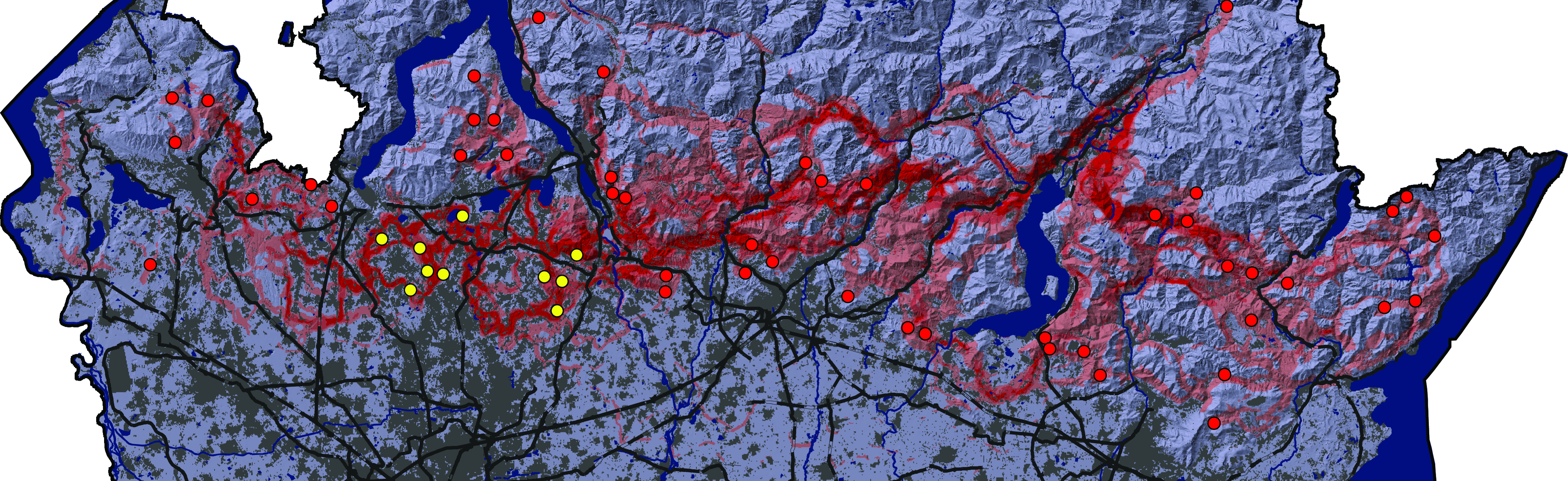

Supplement: Supplementary file 1 — Figure S1. Cumulative map of currents according habitat suitability model with barriers, realized with CIRCUITSCAPE software. [file ece30005-3472-sd1.tif]
